# *Helicobacter pylori* infection reduces the risk of Barrett's esophagus: A meta‐analysis and systematic review

**DOI:** 10.1111/hel.12504

**Published:** 2018-06-25

**Authors:** Bálint Erőss, Nelli Farkas, Áron Vincze, Benedek Tinusz, László Szapáry, András Garami, Márta Balaskó, Patrícia Sarlós, László Czopf, Hussain Alizadeh, Zoltán Rakonczay, Tamás Habon, Péter Hegyi

**Affiliations:** ^1^ Institute for Translational Medicine Medical School University of Pécs Pécs Hungary; ^2^ Institute of Bioanalysis Medical School University of Pécs Pécs Hungary; ^3^ Department of Gastroenterology First Department of Medicine Medical School University of Pécs Pécs Hungary; ^4^ Department of Cardiology First Department of Medicine Medical School University of Pécs Pécs Hungary; ^5^ Department of Hematology First Department of Medicine Medical School University of Pécs Pécs Hungary; ^6^ Department of Pathophysiology Medical School University of Szeged Szeged Hungary

**Keywords:** Barrett's esophagus, esophageal adenocarcinoma, gastroesophageal reflux disease, *Helicobacter pylori*, meta‐analysis, systematic review

## Abstract

**Introduction:**

The prevalence of *Helicobacter pylori* infection (HPI) has been decreasing in developed countries, with an increasing prevalence of Barrett's esophagus (BE) and esophageal adenocarcinoma (EAC) at the same time. The aim of our meta‐analysis was to quantify the risk of BE in the context of HPI.

**Methods:**

A systematic search was conducted in 3 databases for studies on BE with data on prevalence of HPI from inception until December 2016. Odds ratios for BE in HPI were calculated by the random effects model with subgroup analyses for geographical location, presence of dysplasia in BE, and length of the BE segment.

**Results:**

Seventy‐two studies were included in the meta‐analysis, including 84 717 BE cases and 390 749 controls. The overall analysis showed that HPI reduces the risk of BE; OR = 0.68 (95% CI: 0.58‐0.79, *P* < .001). Subgroup analyses revealed risk reduction in Asia OR = 0.53 (95% CI: 0.33‐0.84, *P* = .007), Australia OR = 0.56 (95% CI: 0.39‐0.80, *P* = .002), Europe OR = 0.77 (95% CI: 0.60‐0.98, *P* = .035), and North‐America OR = 0.59 (95% CI: 0.47‐0.74, *P* < .001). The risk was significantly reduced for dysplastic BE, OR = 0.37 (95% CI: 0.26‐0.51, *P* < .001) for non‐dysplastic BE, OR = 0.51 (95% CI: 0.35‐0.75, *P* = .001), and for long segment BE, OR = 0.25 (95% CI: 0.11‐0.59, *P* = .001) in case of HPI.

**Conclusions:**

This extensive meta‐analysis provides additional evidence that HPI is associated with reduced risk of BE. Subgroup analyses confirmed that this risk reduction is independent of geographical location. HPI is associated with significantly lower risk of dysplastic, non‐dysplastic, and long segment BE.

## 1 BACKGROUND

Barrett's esophagus (BE) is the only known precursor for esophageal adenocarcinoma (EAC).^1^ The prevalence of BE and incidence of EAC have been increasing in recent decades^2^ and EAC often is evolved in BE.^1^, ^3^ At the same time, the prevalence of *Helicobacter pylori* infection (HPI) is decreasing in developed countries.^4^

There are multiple individual studies, both with evidence for and against the risk reduction in case of HPI. In 3 of the 4 previous meta‐analyses, HPI proved to reduce the risk of BE.^5^, ^6^, ^7^ On the contrary, Wang et al^8^ did not find a clear relationship between HPI and BE in their analysis. The 3 earlier meta‐analyses used small subsets of studies; they included 5, 9, and 12 trials.^6^, ^7^, ^8^ The most recent and extensive meta‐analysis of Fischbach identified 49 trials with data on the association between HPI and BE. Besides proving the risk reduction, their other main findings were the significant heterogeneity among the studies included and a marked risk reduction in the case of CagA‐positive strains of *H. pylori*. The source of heterogeneity was one of the foci of their discussion and they concluded that both selection and information bias potentially contributed to their results.^5^

The above meta‐analyses have not published analytical results of subgroup analysis for geographical location of the study populations, for the segment length of the BE, and for the presence of dysplasia in BE. Our aim was to update the most recent meta‐analysis which included studies until 2010^5^ and to investigate and quantify the risk of BE in these subgroups.

## 2 METHODS

### 2.1 Protocol

An epidemiological meta‐analysis and systematic review was performed using the Preferred Reporting Items for Systematic Review and Meta‐Analysis Protocols (PRISMA‐P).^9^ The analysis was registered in advance on PROSPERO with registration number CRD42017077509.

### 2.2 Search strategy

A systematic search was conducted in PubMed, EMBASE, and COCHRANE databases, from inception to December 2016. Records were managed by EndNote X7.4, software (Clarivate Analytics, Philadelphia, PA, USA) to exclude duplicates.

PICO items of the strategy were: (P) adult population with BE, (I) past or current HPI, (C) patients without BE, and (O) prevalence of HPI with and without BE.

Keywords for the computer‐aided search were (Barrett's OR Barrett's metaplasia OR Barrett metaplasia OR Barrett's oesophagus OR Barrett's esophagus OR Barrett oesophagus OR Barrett esophagus) AND (*Helicobacter pylori* or *H pylori* or *H. pylori* or *Helicobacter*). Additional articles were identified from the reference lists of eligible primary studies.

### 2.3 Inclusion and exclusion criteria

All studies with relevant information on HPI prevalence in BE patients and controls within the same population were included in our analysis. All studies with abstracts in English were included; full‐text articles in languages other than English were read, appraised, and data were extracted by researchers who spoke and understood the respective language. Full‐text articles and abstracts were both included. Different articles reporting data on the prevalence of HPI (proven by serological and/or histological studies and/or stool antigen testing and/or bacterial culture and/or rapid urease or urea breath test) and BE from the same population were thoroughly scrutinized and only one record with the highest number of BE cases was included in the meta‐analysis. Articles from identical populations where the prevalence of HPI was more detailed for different lengths of BE were excluded from the overall analysis, but they were included in the subgroup analysis for BE segment length. All types of observational studies, such as case control and cross‐sectional studies were included, regardless whether they were prospective or retrospective. Non‐human studies and review articles were excluded.

### 2.4 Data extraction

Numeric data were extracted by 3 investigators and manually populated onto a purpose designed Excel 2016 sheet (Office 365, Microsoft, Redmond, WA, USA). Data were collected on year of publication, study type, geographical location, number of cases and controls, and basic demographics (age, sex ratio) in both groups and method(s) of HPI testing. Most importantly, data were collected on the prevalence of HPI in BE cases and controls, also in dysplastic and non‐dysplastic BE and in different segment lengths of BE, for further subgroup analysis. Data on prevalence of HPI by CagA‐positive strains were also collected. Other relevant findings were mentioned in an additional column as free text. The data extraction was reviewed and conflicts were resolved by the first author.

### 2.5 Statistical analysis

*Helicobacter pylori* infection prevalence data from individual studies were extracted and raw data (number of BE patients with HPI, number of patients without HPI, number of controls with HPI, number of controls without HPI) were calculated, followed by the calculation of odds ratios (ORs) and 95% confidence intervals (CIs) for risk of BE in case of HPI. Adjusted ORs from the original articles were not extracted. Pooled estimates were calculated with random effects model using the DerSimonian‐Laird method.^10^ Results of the meta‐analysis were displayed graphically on forest plots. Heterogeneity was assessed using Cochrane's Q and the *I* ^2^ statistics, where Q exceeds the upper tail critical value of chi‐square on *k*−1 degrees, and *I* ^2^ represents the percentage of effect size heterogeneity that cannot be explained by random chance. As suggested by the Cochrane Handbook, *I* ^2^ values were interpreted as moderate (30%‐60%), substantial (50%‐90%), and considerable (75%‐100%) heterogeneity.^11^ Publication biases of the included studies were checked by Egger's test^12^ and by visual assessment of funnel plots.

All calculations were performed by Stata 11 data analysis and statistical software (Stata Corp LLC, College Station, TX, USA).

### 2.6 Analysis of risk of bias and quality assessment

The assessments of both the risk of bias and the risk of quality were done at the outcome level.

A modified Newcastle‐Ottawa Scale for case control studies was used for the quality assessment of the individual studies included in our meta‐analysis, with the following items, and the result of the assessment was graphically demonstrated in a table with color codes, green: low risk of bias; yellow: moderate or unknown risk of bias; red: high risk of bias.

The questions for the risk assessment were as follows:

1 Was the case definition clear?
  a Yes, with positive endoscopic features of BE and supporting histology (green).
  b Yes, without history of BE (yellow).
  c No clear description of diagnosis of BE (red).
2 Were the BE cases representative?
  a Yes, consecutive BE cases, without significant exclusion criteria (green).
  b No, significant exclusion criteria or no description (red).
3 Was the selection of controls without selection bias?
  a Yes, community controls (green).
  b Hospital controls (endoscopy, blood donors, etc.) (yellow).
  c No clear definition of controls (red).
4 Was the definition of controls clear?
  a Yes, with an endoscopy excluding BE (green).
  b No or no endoscopic exclusion of BE (red).
5 Were the BE cases and controls comparable?
  a Yes, with both age and sex matched (green).
  b Yes, with age or sex (yellow).
  c No (red).
6 Was the investigator blind to the presence of BE, when reading the result of *H. Pylori* test result, or vice versa?
  a Yes, the study description clearly states it.
  b No or no clear description.
7 Was the same method used to test HPI in BE and controls?
  a Yes (green).
  b No or no description (red).

## 3 RESULTS

### 3.1 Study selection

**Figure 1:**
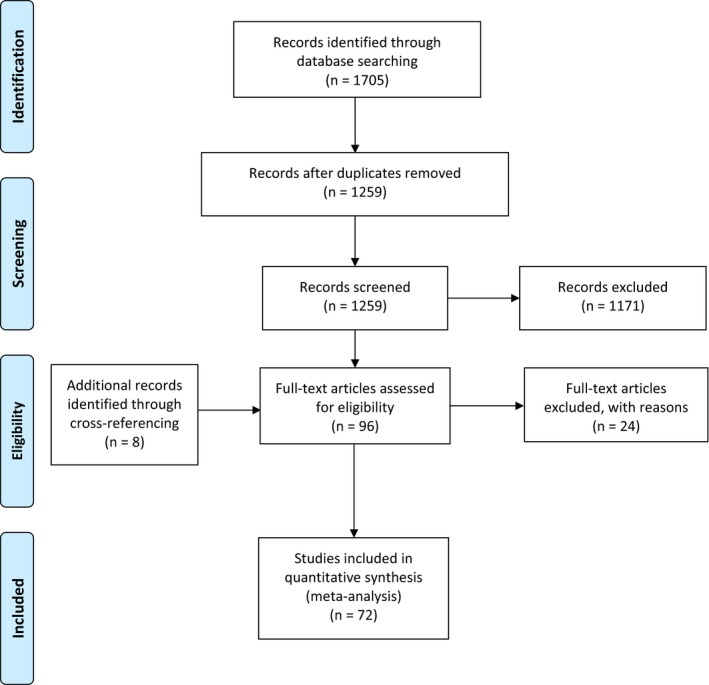
Flow chart of the study selection process

Our search strategy initially identified 1705 potential studies. Removal of duplicates was followed by screening first the titles, and then the abstracts, leaving 96 studies for full‐text review, including 8 additional studies identified in the reference lists of the primary eligible studies.^13^, ^14^, ^15^, ^16^, ^17^, ^18^, ^19^, ^20^ Thirteen studies were excluded, as they did not provide sufficient data (reasons for exclusion detailed in Appendix S1). Data were extracted from 83 studies^13^, ^14^, ^15^, ^16^, ^17^, ^18^, ^19^, ^20^, ^21^, ^22^, ^23^, ^24^, ^25^, ^26^, ^27^, ^28^, ^29^, ^30^, ^31^, ^32^, ^33^, ^34^, ^35^, ^36^, ^37^, ^38^, ^39^, ^40^, ^41^, ^42^, ^43^, ^44^, ^45^, ^46^, ^47^, ^48^, ^49^, ^50^, ^51^, ^52^, ^53^, ^54^, ^55^, ^56^, ^57^, ^58^, ^59^, ^60^, ^61^, ^62^, ^63^, ^64^, ^65^, ^66^, ^67^, ^68^, ^69^, ^70^, ^71^, ^72^, ^73^, ^74^, ^75^, ^76^, ^77^, ^78^, ^79^, ^80^, ^81^, ^82^, ^83^, ^84^, ^85^, ^86^, ^87^, ^88^, ^89^, ^90^, ^91^, ^92^, ^93^, ^94^, ^95^; however, 11 of these studies had to be excluded from the statistical analysis as they contained data from same populations already described in other articles.^22^, ^35^, ^38^, ^39^, ^52^, ^56^, ^65^, ^68^, ^80^, ^81^, ^82^ Our final statistical analysis included 72 studies. Of the 72 articles, 2 studies contained data from populations already reported in the 70 studies; however, these had detailed data on the different prevalence of HPI in different segment lengths of BE, therefore these were only included in the subgroup analysis.^45^, ^84^ The study selection process is shown in Figure 1. The summary of the characteristics of the studies included in our analysis is shown in Table 1.

**Table 1:** Main characteristics of the studies included

Study author and year	Country	Number of cases/controls	*Helicobacter pylori* testing method	Definition of controls	Only new BE cases
Abbas (1995)^21^	Pakistan	29/29	H, R	GERD	No
Abe (2009)^23^	Japan	36/108	H, R, S	Population	Yes
Abouda (2003)^13^	UK	60/25	H, R, S	Endoscopy	No
Ackermack (2003)^24^	Netherlands	51/62	S	Endoscopy	Not stated
Ahmed (2004)^25^	Sudan	11/47	R	GERD	Not stated
Anderson (2008)^26^	Ireland	224/260	S	Population	Yes
Blaser (1991)^27^	USA	58/41	H,S	Population	Not stated
Carmona (2003)^28^	Mexico	24/232	R	Endoscopy	Not stated
Chacaltana (2009)^14^	Peru	11/911	H	Other	No
Chang (2010)^29^	China	32/41	H	Endoscopy	No
Chen (2016)^30^	Taiwan	161/644	R	Endoscopy	Not stated
Cooper (1991)^31^	UK	26/30	H	GERD	No
Corley (2008)^32^	USA	318/299	S	Population	Yes
Csendes (1997)^33^	Chile	100/190	H	Endoscopy	No
Dore (2016)^34^	Italy	131/1772	H, R, U	Endoscopy	No
El Serag (1999)^15^	USA	36/72	H	GERD	No
Fassan (2009)^36^	Italy	210/210	H	Endoscopy	Not stated
Ferrandez (2006)^37^	Spain	104/213	H, R, S, PCR	Population	No
Goldblum (2002)^40^	USA	70/60	H, S	Endoscopy	No
Hackelsberger (1998)^41^	Germany	16/315	H, R	Endoscopy	No
Henihan (1998)^42^	Ireland	82/40	H esophagus	GERD	No
Hilal (2016)^43^	USA	323/1849	H	Endoscopy	No
Hirota (1999)^44^	USA	104/738	H esophagus	Endoscopy	No
Inomata (2006)^a^, ^45^	Japan	36/80	H, R, S	Endoscopy	Not stated
Johansson (2007)^46^	Sweden	21/498	H esophagus	Endoscopy	No
Jonaitis (2011)^47^	Lithuania	33/160	H, R	GERD	Not stated
Kala (2007)^16^	Czech Rep.	22/173	H, R	GERD	No
Katsienlos (2013)^48^	Greece	75/1915	H, R	Endoscopy	Not stated
Keyashian (2013)^49^	USA	52/391	H, SA	Endoscopy	No
Kiltz (1999)^50^	Germany	35/320	R, S	Endoscopy	No
Kim (2006)^51^	S. Korea	31/224	H, R	Endoscopy	Not stated
Laheij (2002)^53^	Netherlands	23/528	H, R, C	Endoscopy	Not stated
Lam (2008)^54^	USA	56/280	S	Endoscopy	Yes
Lee (2011)^55^	Malaysia	15/104	H, R	Endoscopy	Not stated
Loffeld (1992)^57^	Netherlands	71/200	H esophagus, S	Population	Not stated
Loffeld (2000)^58^	Netherlands	36/454	H	Endoscopy	Yes
Loffeld (2004)^59^	Netherlands	307/5341	H, C	Endoscopy	No
Lord (2000)^60^	Australia	91/214	H	Endoscopy	No
Martinek (2003)^61^	Czech Rep.	31/259	H, R	Endoscopy	Not stated
Meng (2008)^17^	USA	28/104	PCR	Endoscopy	Not stated
Monkemuller (2008)^62^	Germany	97/97	H	Endoscopy	No
Nandurkar (1997)^63^	Australia	46/112	H esophagus	Endoscopy	Yes
Newton (1997)^64^	UK	16/25	H, R	Endoscopy	No
Pascareno (2014)^66^	Romania	24/218	H	Endoscopy	Not stated
Paull (1988)^67^	USA	26/26	H	Endoscopy	No
Peng (2009)^69^	China	27/110	R	GERD	Not stated
Rajendra (2004)^18^	Malaysia	123/1741	H, R	Endoscopy	Not stated
Rajendra (2007)^70^	Malaysia	55/53	H, S	Endoscopy	No
Rex (2003)^71^	USA	48/764	R	Population	Yes
Rodriguez (2014)^72^	Spain	8/192	H	Endoscopy	Yes
Ronkainen (2005)^19^	Sweden	16/984	H, C, S	Population	Not stated
Rubenstein (2014)^73^	USA	150/177	S	Endoscopy	No
Rugge (2001)^74^	Italy	53/53	H	Endoscopy	Not stated
Sharifi (2014)^76^	Iran	34/702	H, R	GERD	Not stated
Schenk (1999)^75^	Netherlands	49/88	H	GERD	No
Sonnenberg (2010)^77^	USA	2510/76 475	H	Endoscopy	No
Sonnenberg (2016)^78^	USA	76 475/284 552	H	Endoscopy	No
Thrift (2012)^79^	Australia	0/398	S	Population	Yes
Toruner (2004)^20^	Turkey	29/306	H	Endoscopy	Yes
Uno (2011)^83^	Japan	126/100	H, S, R	Endoscopy	No
Vaezi (2000)^a^, ^84^	USA	83/60	H, S	GERD	Not stated
Veldhuyzen (2006)^85^	Canada	25/1015	H	Endoscopy	Yes
Vicari (1998)^86^	USA	48/57	H,S	GERD	No
Vieth (2000)^87^	Germany	1054/712	H	Endoscopy	No
Watari (2009)^88^	Japan	88/52	H, C	Other	No
Werdmuller (1997)^89^	Netherlands	13/399	H, C, R, S	Endoscopy	Not stated
Weston (2000)^90^	USA	208/217	H	GERD	No
White (2008)^91^	Canada	39/29	H esophagus	Endoscopy	No
Wong (2002)^92^	China	10/448	H, R, U	Endoscopy	Yes
Wu (2000)^93^	Hong Kong	6/85	H, R	GERD	Not stated
Zaninotto (2002)^94^	Italy	34/32	H esophagus	GERD	No
Zullo (2014)^95^	Italy	17/1037	H	Endoscopy	Not stated

C, culture; GERD, gastro‐esophageal reflux disease; H, histology; PCR, polymerase chain reaction; R, rapid urease test; S, serology; SA, stool antigen; U, urea breath test.

a Studies only in the subgroup analysis for BE segment length.

These studies are indicated as H esophagus in column 4 (H. pylori testing method).

### 3.2 Results of statistical analysis

#### 3.2.1 Risk of Barrett's esophagus in case of *Helicobacter pylori* infection

Our results confirmed an overall risk reduction OR = 0.68 (95% CI: 0.58‐0.79, *P* < .001) by the calculation from the data of the 70 studies, including a total of more than 90 000 BE cases and nearly 400 000 controls. Heterogeneity was substantial, *I* ^2^ = 84.0%.

**Figure 2:**
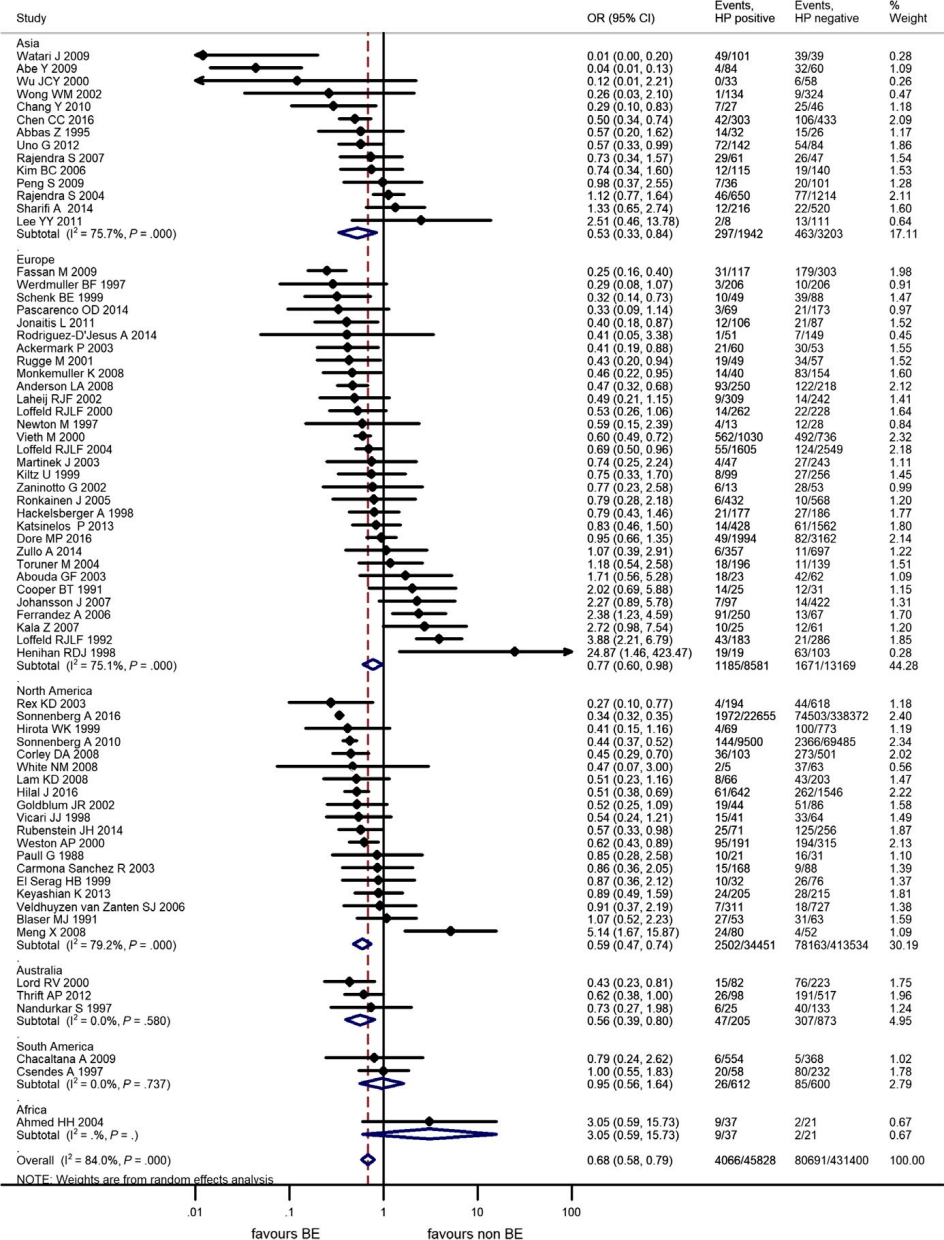
Forest plot of the random effect analysis of the 70 studies included in the overall analysis, in subgroups for continents. OR, odds ratio; CI, 95% confidence interval; HP,* Helicobacter pylori*, weights of studies and heterogeneities are indicated too

Subgroup analyses showed risk reduction in Asia, OR = 0.56 (95% CI: 0.35‐0.90, *P* = .016), 14 studies; in Australia, OR = 0.56 (95% CI: 0.39‐0.80, *P* = .002), 3 studies; in Europe, OR = 0.75 (95% CI: 0.58‐0.96, *P* = .022), 31 studies, and in North‐America, OR = 0.59 (95% CI: 0.47‐0.74, *P* < .001) 19 studies. The low number of studies with considerable selection and information bias from South‐America^14^, ^33^ and Africa^25^ means that the meta‐analytical calculations of the studies from these regions are not suitable for any conclusions, although these studies could not demonstrate a clear association between HPI and BE. Detailed results from the 70 studies are detailed in Figure 2.

#### 3.2.2 Risk of Barrett's esophagus in case of CagA‐positive *Helicobacter pylori* infection

There were 4 additional studies reporting the prevalence of CagA‐positive HPI in relation to BE, in addition to the studies identified by Fischbach et al^5^ which included results from 7 studies. In total, 11 studies were included in the subgroup analysis.^17^, ^24^, ^26^, ^32^, ^37^, ^58^, ^70^, ^73^, ^74^, ^84^, ^86^ A further study from Abouda et al in 2003 reported data on *H. pylori* strain positive for both CagA and VacA and not CagA strains only. As their data reported on a more specific *H. pylori* strain, their results were not included in our subgroup analysis.^13^ The calculated risk reduction for BE in case of CagA‐positive HPI is significant, OR = 0.50 (95% CI: 0.29‐0.87, *P* = .014). Fischbach et al calculated an OR = 0.38 (95% CI: 0.189‐0.781), and our result confirms their finding. The forest plot of this subgroup analysis is shown in Figure S1.

#### 3.2.3 Risk of different segment lengths of Barrett's esophagus in case of *Helicobacter pylori* infection

Prevalence of HPI for different segment lengths of BE was detailed in 7 studies and data were suitable for meta‐analysis.^44^, ^45^, ^47^, ^66^, ^70^, ^71^, ^83^, ^84^, ^94^ Two articles had detailed data on ultrashort segment BE (USSBE, <1 cm)^66^, ^94^ and they were not included in the short segment BE (SSBE) subgroup. We note that the new guideline of the British Society of Gastroenterology defines BE by at least 1 cm of metaplastic columnar lining, which questions the justification of the diagnosis of USSBE.^96^ However, the meta‐analytical calculation was performed for this subgroup as well.

**Figure 3:**
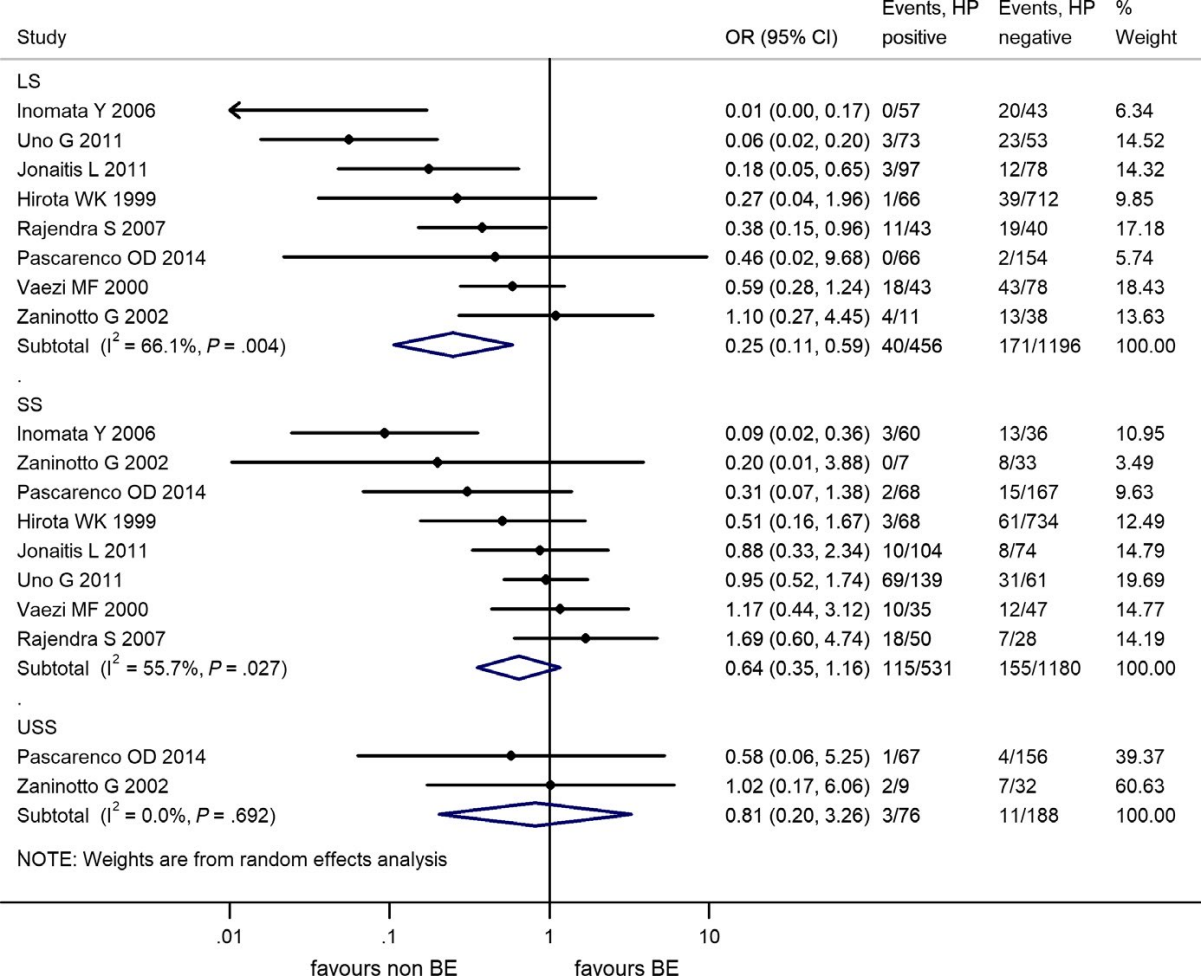
Forest plot of the random effect analysis of the 7 studies included in the subgroup analysis for different segment lengths of Barrett's esophagus. LS, long segment; SS, short segment; USS, ultrashort‐segment Barrett's esophagus; OR, odds ratio, CI, 95% confidence interval; HP,* Helicobacter pylori*, weights of studies and heterogeneities are indicated too

The risk reduction was statistically significant in the long segment BE (LSBE) subgroup OR = 0.25 (95% CI: 0.11‐0.59, *P* = .001). In SSBE, the pooled OR was not statistically significant, but there is likely a risk reduction, OR = 0.63 (95% CI: 0.32‐1.26, *P* = .191). The results on USSBE or intestinal metaplasia at the cardia are not suitable for any conclusion, but there does not seem to be a reduced risk. The results are detailed in Figure 3.

#### 3.2.4 Risk of dysplasia in Barrett's esophagus in case of *Helicobacter pylori* infection

Prevalence of HPI in association with the presence of dysplasia in BE was detailed in 7 studies and data were suitable for meta‐analysis.^36^, ^42^, ^78^, ^79^, ^86^, ^87^, ^90^ We defined the subgroup of dysplastic BE by the presence of low‐ or high‐grade dysplasia or adenocarcinoma in the BE.

The risk reduction was significant for dysplastic BE in case of HPI, OR = 0.37 (95% CI: 0.26‐0.51, *P* < .001). We note that the study by Henihan et al^42^ did not report any dysplastic BE with HPI; therefore, the result of their study could not be interpreted by the random effect model in this subgroup and had to be excluded.

**Figure 4:**
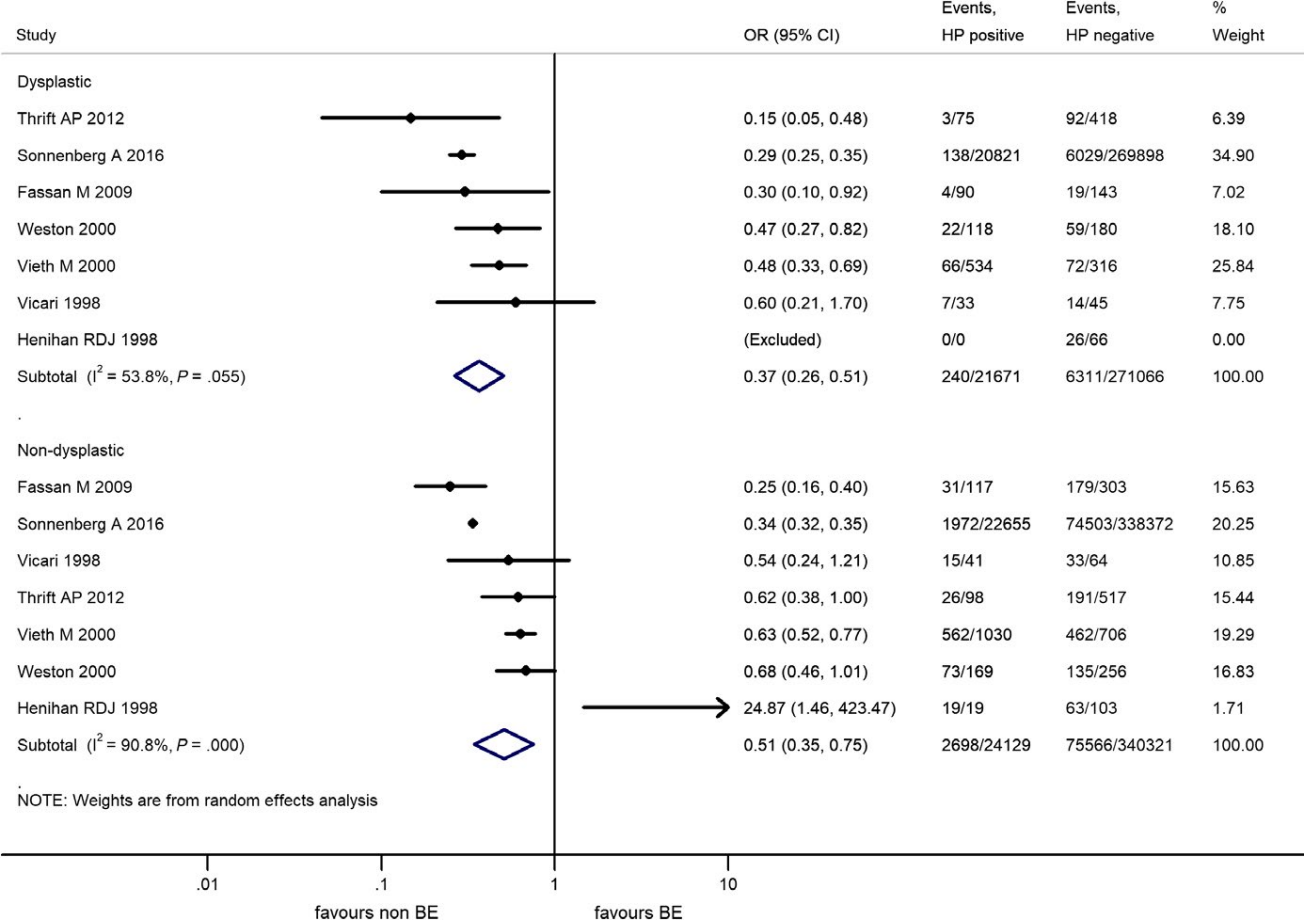
Forest plot of the random effect analysis of the 6 studies included in the subgroup analysis for the presence of dysplasia in Barrett's esophagus. OR, odds ratio; CI, 95% confidence interval; HP,* Helicobacter pylori*, weights of studies and heterogeneities are highlighted too

In non‐dysplastic BE, the risk reduction was also significant, OR = 0.51 (95% CI: 0.35‐0.75, *P* = .001). The results are detailed in Figure 4.

#### 3.2.5 Additional subgroup analyses to identify the source of heterogeneity

In order to understand the association between the risk of BE and the prevalence of HPI, further subgroup analyses were performed.

Stratification by the different control groups was possible for 4 subgroups of studies with population, gastro‐esophageal reflux disease (GERD), endoscopy, and other controls as indicated in Table 1. In subgroups of studies with population and GERD controls, the ORs were not significant. Only the studies with endoscopy controls showed a significant risk reduction OR = 0.48 (95% CI: 0.31‐0.74, *P* = .001). There was substantial and considerable heterogeneity among studies in all subgroups. The detailed results are shown in Figure S2.

Stratification by the *H. pylori* testing method was possible for 4 subgroups of studies with histology from the stomach, histology from the esophagus, serology, and rapid urease test as indicated in Table 1. One study used polymerase chain reaction and in 30 studies multiple modalities were used for the detection of HPI. In case of rapid urease test and histology from the esophagus, the ORs from the studies cover a wide range and the pooled ORs for these methods are not significant. Significant risk reduction was seen in the pooled ORs for *H. pylori* testing by histology from the stomach and serology. Heterogeneities in all subgroups are substantial, save for serology where the studies showed no significant heterogeneity (*I* ^2^ = 0%, *P* = .906). The detailed results are shown in Figure S3.

We identified 12 studies, which clearly stated that only new Barrett's cases were included or previously diagnosed BE cases were excluded.^20^, ^23^, ^26^, ^32^, ^54^, ^58^, ^63^, ^71^, ^72^, ^79^, ^85^, ^92^ The subgroup analysis showed an OR = 0.48 (95% CI: 0.34‐0.68, *P* < .001) with substantial heterogeneity (*I* ^2^ = 60.6%, *P* = .003). The detailed results are shown in Figure S4.

#### 3.2.6 Risk of publication bias

The Egger's tests calculated significant publication bias in the meta‐analysis of all 70 studies, *P* < .001, but not in the subgroup analyses on the CagA status (*P* = .188), the segment length of BE (*P* = .051), the presence of dysplasia (*P* = .16), and the newly diagnosed BEs (*P* = .465).

A visual inspection of the funnel plot of the overall assessment from the 70 studies revealed asymmetry, Figure S5. There was no asymmetry on the funnel plots of the subgroup analyses.

#### 3.2.7 Risk of selection and information bias

The results of our quality and risk assessment by the modified Newcastle‐Ottawa scale for case control studies are shown in Table 2.

**Table 2:**
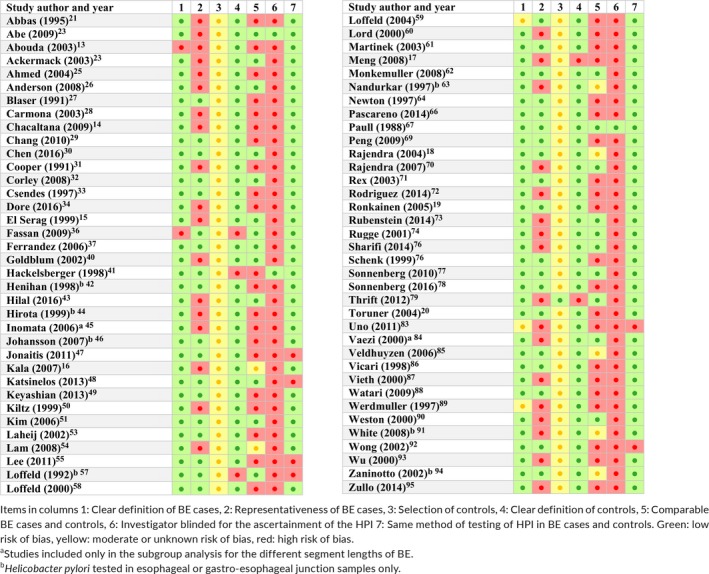
Results of the modified Newcastle‐Ottawa quality assessment scale for case control studies

It is important to note that our meta‐analysis includes 78 studies of the meta‐analysis by Fischer and our quality and risk assessment revealed both selection and information bias, which had been reported by Fischbach et al^5^ In‐depth scrutiny for causes of the bias in the additional 25 studies showed a similar pattern of flaws in the study design.

1. Lack of clear definition of BE. Although most of the studies defined it by endoscopy and histology findings at the same time, these diagnostic criteria show variability in time and place.
2. The BE cases included in the studies were often limited by many exclusion criteria.
3. We found only one study in which the controls truly represented the population^79^; most of the other studies used endoscopy controls. A smaller proportion of studies used blood donors as controls, who are often healthier and younger than the normal population.
4. Selection of controls in endoscopy is necessary in the exclusion of asymptomatic Barrett's patients from the controls, but it means that these controls go through a gastroscopy with the purpose of investigating gastrointestinal symptoms, which most likely influences their prevalence of HPI even if there is no gastritis or ulcer disease. Patients with gastro‐esophageal reflux disease (GERD) formed the control group in several studies. This also results in bias, as there is convincing evidence that HPI reduces the risk of GERD.^6^
5. Comparability was poor in most of the studies, as only 23 of the studies had age‐ and sex‐matched cases and controls and an additional 7 of them had either sex‐ or age‐matched cases and controls. Some of the studies described significantly different proportion of races in the cases and controls and there is ample evidence that ethnicity influences the prevalence of both HPI and BE.^49^, ^54^, ^65^
6. Only 3 studies stated clearly that the investigators were blinded to BE when testing HPI or vice versa. In some of the articles, the study design suggested that the single pathologist involved was obviously aware of the BE and the HPI status when assessing the histology slides for BE and HPI, while in other studies the endoscopist was aware of the BE diagnosis at the time when the rapid urease test was performed. However, the vast majority of the studies did not describe the process of HPI ascertainment; this is also a potential source of bias.
7. Testing of HPI in the studies was performed by the same method in both groups in nearly all studies. However, some articles described alternative methods of HPI testing (ie positive result of rapid urease test and/or histology and/or culture and/or serology and/or stool antigen test) and it is not clear what proportion of these tests were used in the cases and controls.

## 4 DISCUSSION

### 4.1 Strengths of the analysis

To date, this is the largest and most comprehensive meta‐analysis in this topic and includes data from 5 continents and 72 individual studies. To our best knowledge, this is the first meta‐analysis on the effect of HPI on the length of BE and the presence of dysplasia in BE.

### 4.2 Limitations of the analysis

Due to the limitations of the studies, the inconsistency of results, the indirect nature of the evidence, and the imprecision and reporting bias, the grade of evidence is low at best, based on the Grading of Recommendations Assessment, Development and Evaluation (GRADE) tool. Therefore, further research on this topic would very likely have an impact.^97^

### 4.3 Heterogeneity among the studies

Our subgroup analyses for geography, CagA status, segment length of BE, dysplastic BE, control groups, *H. pylori* testing method, and new diagnoses of BE revealed substantial and in cases considerable heterogeneity among the studies, apart from 3 subgroups in all analytical calculations.

There was no heterogeneity among studies from Australia, South America, and from studies where serology was used to detect HPI. In the subgroup of Australia and South America, the small subsets of studies, 3 and 2 respectively, caution us to conclude that geography accounts for heterogeneity. However, subgroup analyses with smaller or more accurately specified geographic areas could show different results.

There were 6 studies in the subgroup with serology as the method of detection of HPI.^24^, ^26^, ^32^, ^54^, ^73^, ^79^ The studies are from 2003 to 2014, 3 studies from the USA, 1 from Netherlands, Ireland, and Australia, 3 studies with population, and 3 studies with endoscopy controls. Four of the studies used IgG enzyme assays, 1 western blot, and 1 did not specify the exact method. None of the testing enzyme assays were the same. However, the homogeneity among these studies suggests that risk stratification of BE by HPI status could be best assessed by a serological test.

### 4.4 Potential explanations of our findings

The role of *H. pylori* in the pathogenesis of BE is often described as controversial.^98^ As mentioned before, our meta‐analysis showed an inverse association between HPI and BE; however there are several previous studies with altogether different conclusions: reporting that HPI has no correlation with BE^33^, ^69^ or even a positive association^42^, ^57^ (describing HPI as a risk factor). Most papers (especially the ones with higher patient numbers) are in parallel with our findings.^32^, ^36^, ^77^

If we accept that HPI leads to risk reduction, the following question arises: What could be the cause or mechanism behind this inverse association? This question is not only important from a theoretical, but also from a clinical standpoint: understanding the mechanism is crucial for evaluating the risks and benefits of *H. pylori* eradication therapy, in addition to bringing us closer to explaining the increasing incidence of BE.

*Helicobacter pylori* infection is a proven risk factor for gastric non‐cardia adenocarcinoma and other cancers including lymphoma; however not much is known about its relationship with gastric cardia and EAC.^99^ Epidemiological data show a simultaneous decline of HPI and increase in the aforementioned 2 tumor types. Along with the decrease of *H. pylori* positivity, the incidence of non‐cardia adenocarcinomas is also falling.^100^

As to why and how exactly could HPI reduce the risk of BE development, several theories exist, but none of them are considered proven. Multiple articles attribute this fact to the effect of *H. pylori* on the gastric mucosa: the microorganism causes a corpus‐predominant gastritis, which leads to decreased gastric output. In this case, the esophagus is less exposed to the harmful effect of gastric acid, thus it has a reduced risk for developing BE and EAC.^5^, ^7^, ^98^, ^101^

Several studies that did not find a negative correlation between HPI and BE only did so when looking at patients that were infected with a CagA‐positive subgroup *of H. pylori*.^99^ Other articles that found an inverse association between *H. pylori* and BE reported an even stronger correlation when comparing only the CagA‐positive subgroup instead of all *H. pylori‐*positive patients.^5^, ^7^, ^84^

Chow et al and Vaezi et al hypothesize that this phenomenon might be caused by the CagA‐positive sting's increased virulence toward gastric mucosa and results in a multifocal atrophic gastritis that also involves the destruction of gastric parietal cells, which further impairs acid secretion (more severely as compared to the CagA‐negative subgroup). Consequently, the reduced acidity of the reflux's convent reduces the risk of complications of GERD, such as BE and EAC.^84^, ^99^

Contrary to this theory, based on a population‐based Swedish case control study, Ye et al speculates that it is unlikely that *H. pylori* lowers the risk of BE through the reduction of gastric acidity. They drew this conclusion because no correlation was found between gastric atrophy and EAC in their study; however, they did find a significant association between gastric atrophy and cardia adenocarcinoma.^102^

In a meta‐analysis on the subject, Fischbach et al describes another theory that aims to explain the inverse relationship between *H. pylori* and BE. They speculate that HPI is associated with reduced risk for obesity, thus not only reducing the likeliness for acidic reflux, but also the insulin level in the blood. This leads to the decreased production of Insulin‐like Growth Factor (IGF), which normally acts as an agent that potentiates the proliferation of Barrett's epithelium.^5^ With the reduced amount of circulating IGF due to *H. pylori*, BE is less likely to develop.^103^

In contrast to these theories, Kountouras et al highlighted the conflicting nature of data available on this topic via editorial letters written in response to some previously cited articles. He mentions that in the Malay population, *H. pylori* incidence is traditionally low; however, contrary to expectations, the incidence of BE and distal esophageal tumors is also below average.^104^

He not only points to the fact that according to several studies *H. pylori* might be a risk factor for BE, but also describes potential mechanisms to explain this positive connection. He states that *H. pylori*‐induced overproduction of gastrin contributes to neoplastic progression in Barrett's through pathway signaling. Furthermore, *H. pylori* also has a pro‐inflammatory effect that might also potentiate the said progression.^105^

According to our results and the majority of conclusions available in the literature, a persistent HPI would be desirable for the prevention of BE. However, it is exactly the aforementioned atrophic gastritis that acts as the main risk factor for gastric non‐cardia adenocarcinoma. This 2‐sided effect of *H. pylori* is what causes clinicians to pose the question that is penitently described as Hamletic by Zullo et al^101^: to eradicate or not to eradicate? However, we have to emphasize that there is no evidence that should prevent us from eradicating *H. pylori,* regardless of coexisting reflux esophagitis or BE. HPI needs treatment, when it is identified.

An editorial in Gastroenterology elaborates on the possibility that the decline in *H. pylori* incidence might have other consequences, not necessarily limited to the field of gastroenterology. For example, *H. pylori* might have an effect in regulating ghrelin and leptin, 2 hormones produced (partly in case of leptin) by the stomach and related to metabolism regulation. The article suggests that with the continuous fall of *H. pylori* incidence, we might see an increase in diabetes and obesity due to the dysregulation of these hormones.^100^

Our results confirm the conclusion of previous meta‐analyses^5^, ^6^, ^7^ and we calculated a similar magnitude of risk reduction. Gisbert et al^6^ in 2002 calculated an OR = 0.60 (95% CI: 0.48‐0.76), Rokkas et al^7^ in 2007 found an OR = 0.64 (95% CI: 0.43‐0.94, *P* = .025), and Fischbach et al^5^ in 2012 reported a RR = 0.73 (95% CI: 0.66‐0.80).

In summary, HPI is associated with a reduced risk of BE. Our new findings prove that the risk reduction in case of HPI seems to be independent of the geographical location and it is directly associated with the length of the BE segment and the presence of dysplasia in BE.

## COMPETING INTEREST

No competing interests declared.

## AUTHOR CONTRIBUTIONS

Erőss B and Hegyi P designed the research and the study concept; Sarlós P, Szapáry L, and Tinusz B performed data extraction; Erőss B checked the data extracted, Farkas N analysed and interpreted the data; Erőss B and Tinusz B performed quality and risk assessment, Erőss B, Tinusz B, Farkas N, and Hegyi P wrote the article; Vincze Á, Sarlós P, Garami A, and Balaskó M supervised the study; Czopf L, Alizadeh H, Rakonczay Z, and Habon T conducted a critical revision of the manuscript for important intellectual content; all of the co‐authors granted final approval of the version of the article to be published.
