# Supplementary figures and images for: Helicobacter pylori infection reduces the risk of Barrett's esophagus: A meta‐analysis and systematic review
Source: Helicobacter. 2018 Jun 25;23(4):e12504. doi: 10.1111/hel.12504 (PMC6055671; doi:10.1111/hel.12504)

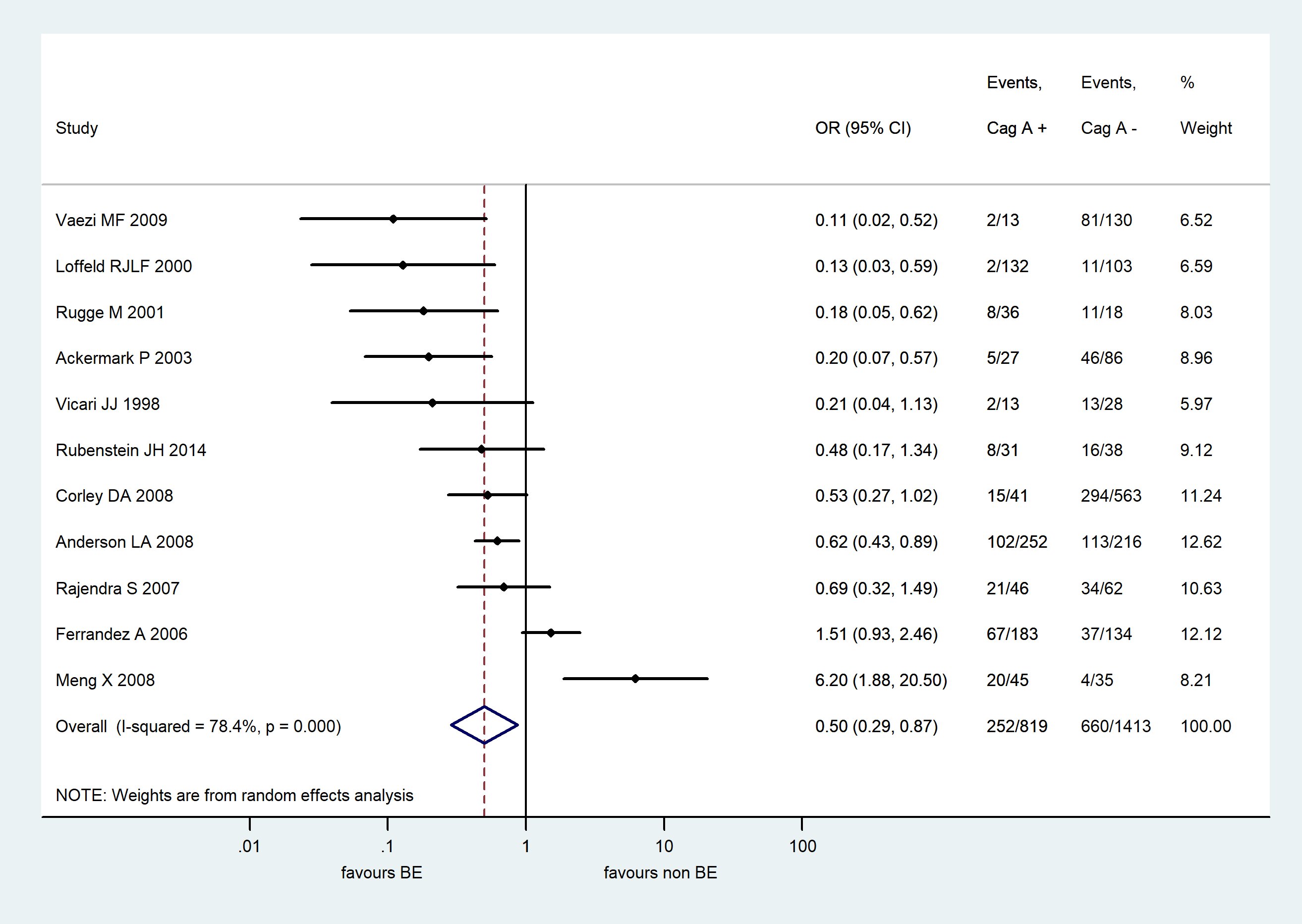

Supplement: Supplementary file 1 [file HEL-23-na-s001.tif]

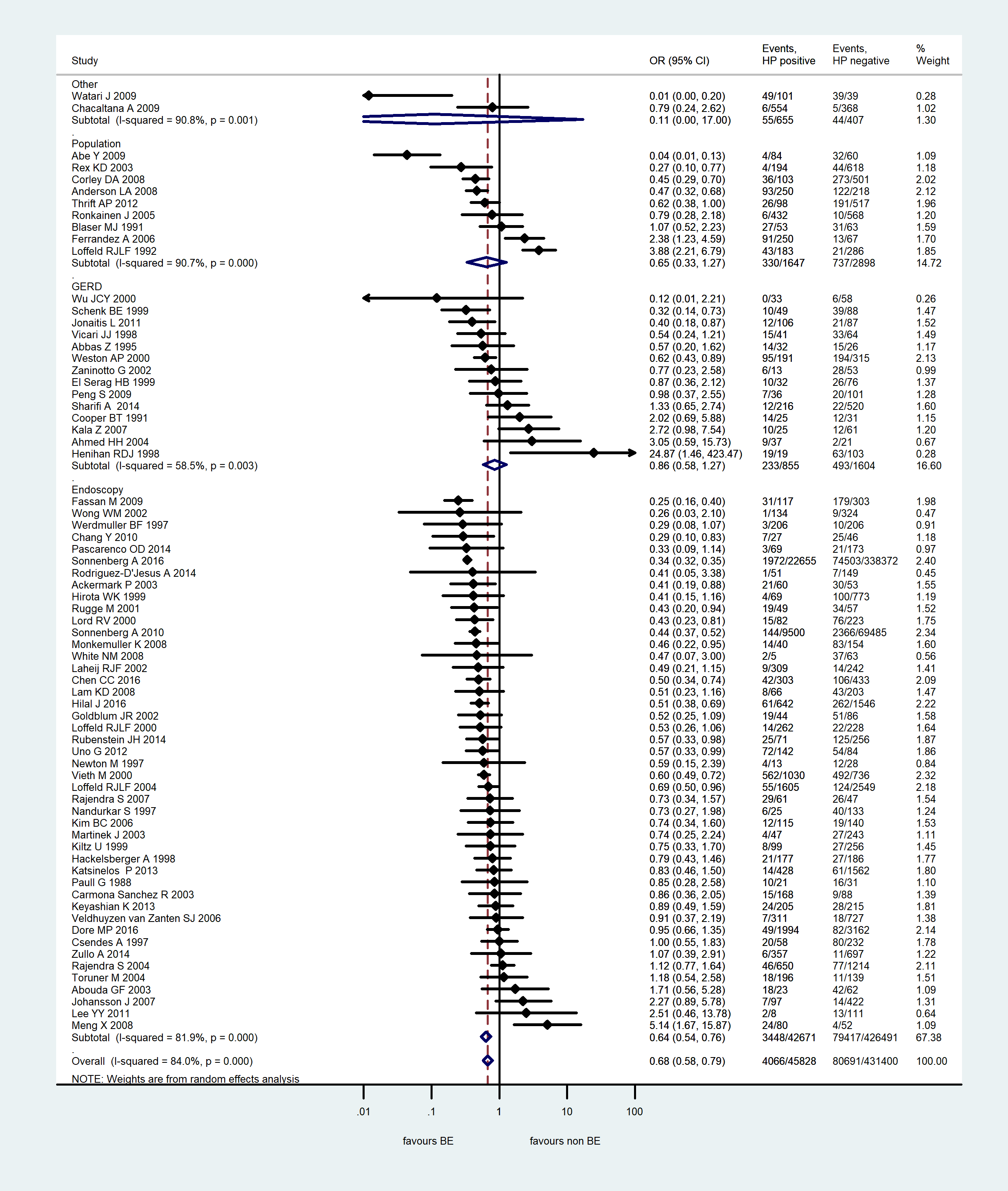

Supplement: Supplementary file 2 [file HEL-23-na-s002.tif]

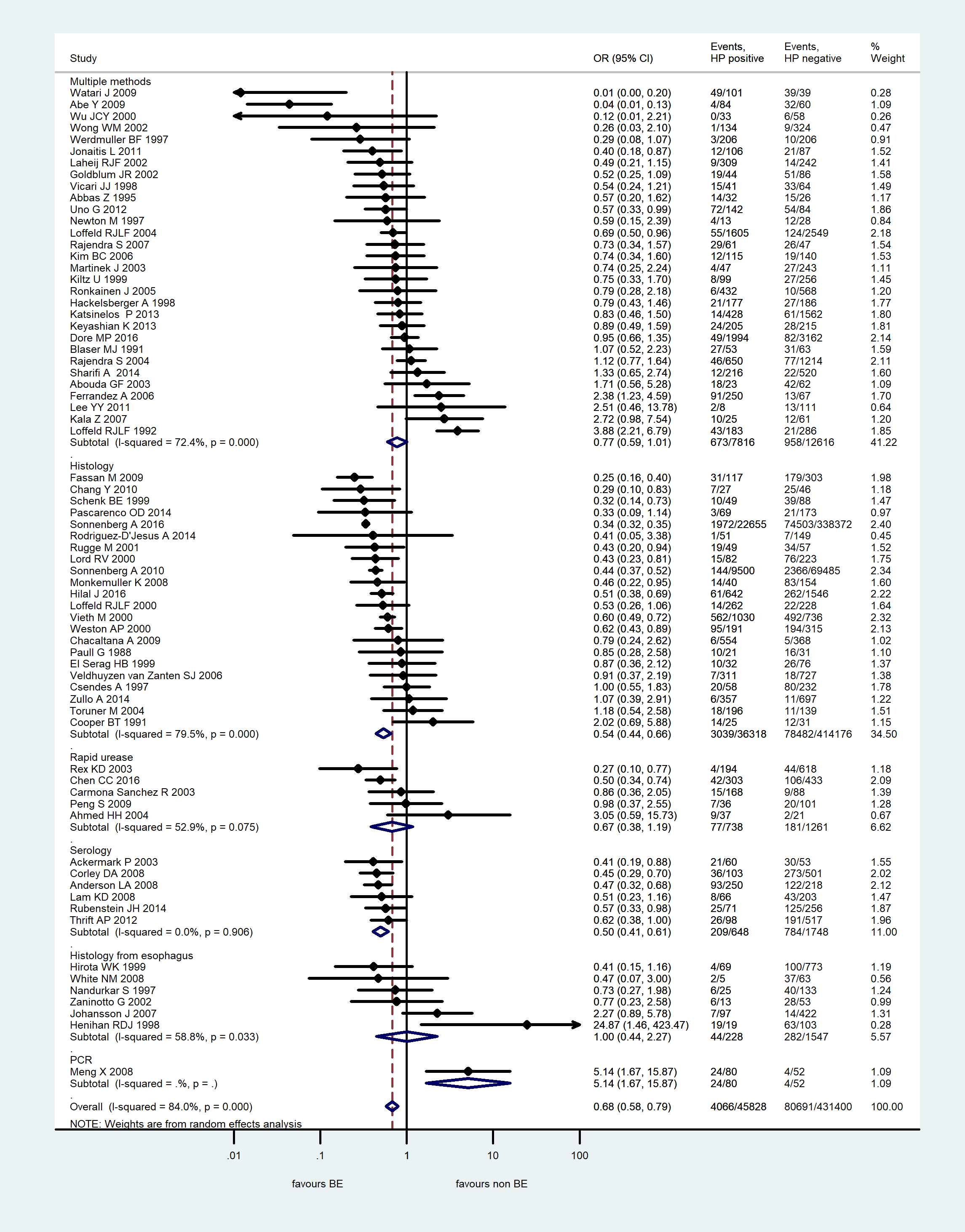

Supplement: Supplementary file 3 [file HEL-23-na-s003.tif]

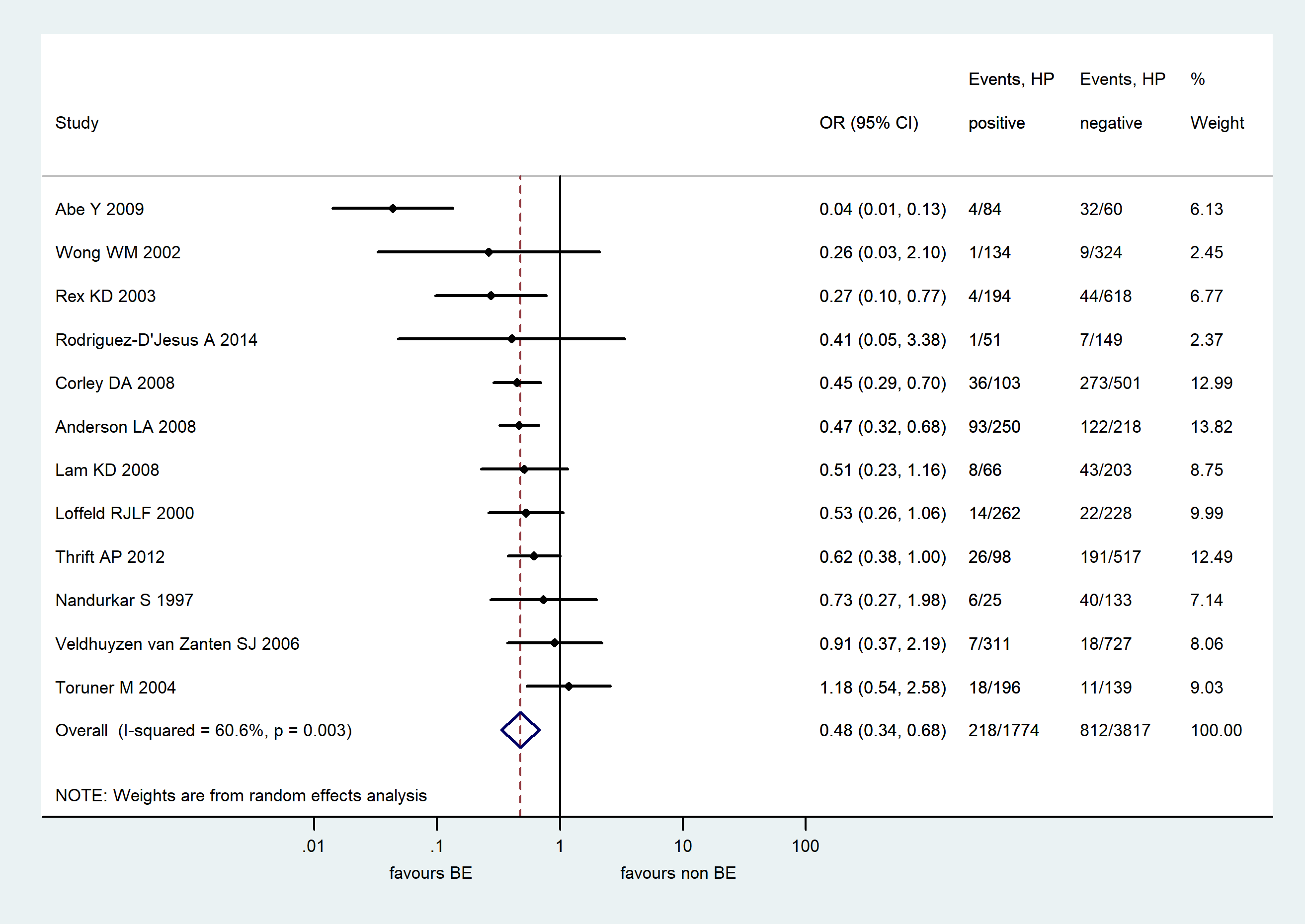

Supplement: Supplementary file 4 [file HEL-23-na-s004.tif]

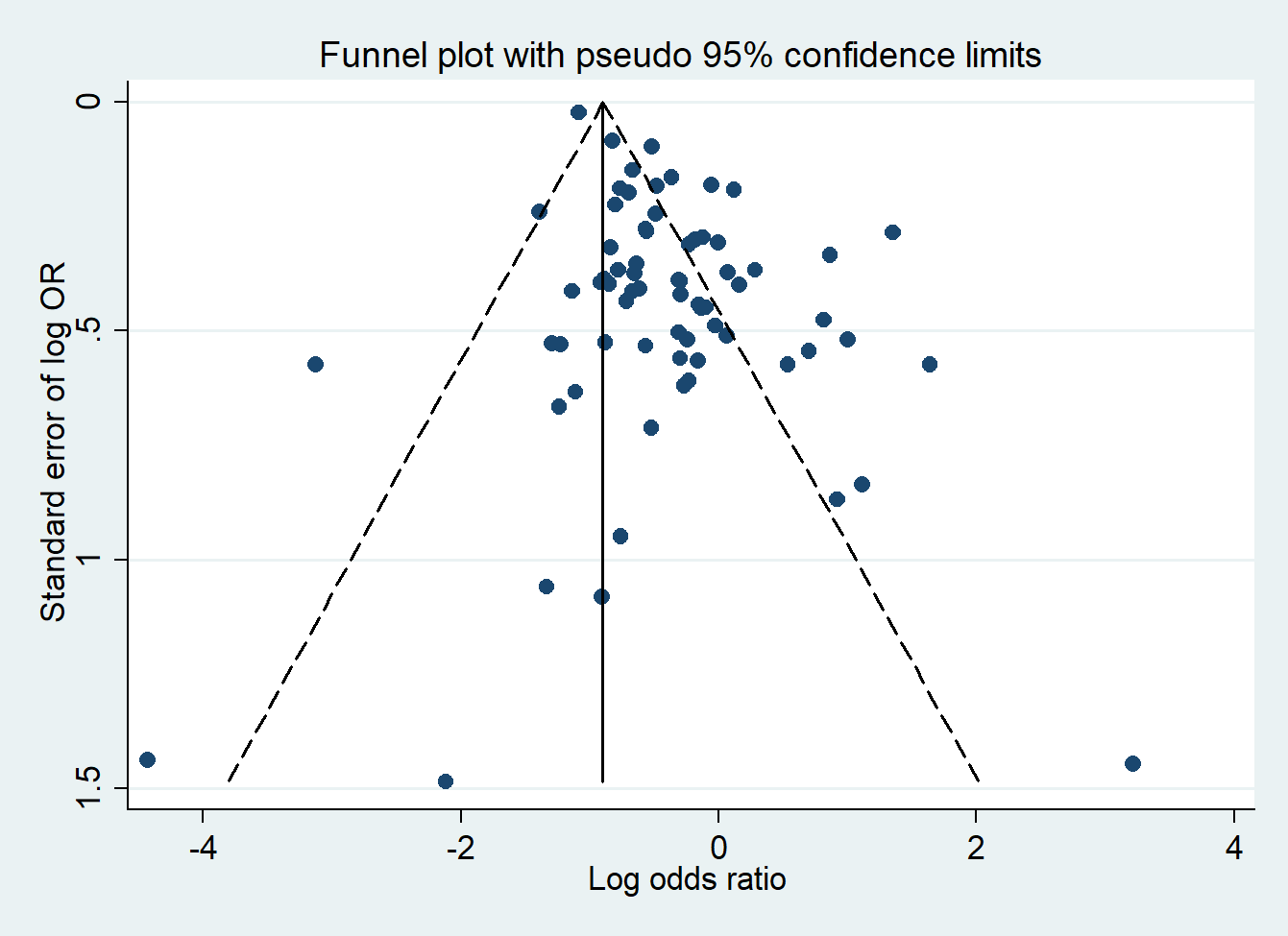

Supplement: Supplementary file 5 [file HEL-23-na-s005.tif]
