# Supplementary material for: Helicobacter pylori infection reduces the risk of Barrett's esophagus: A meta‐analysis and systematic review
Source: Helicobacter. 2018 Jun 25;23(4):e12504. doi: 10.1111/hel.12504 (PMC6055671; doi:10.1111/hel.12504)
Supplement: Supplementary file 6 [file HEL-23-na-s006.docx]

**Reasons for exclusion of 13 studies after full text review.**

**(Reasons other than studies with results from previously published populations.)**

Agreus et al from Sweden reported prevalence of HPI only in reflux patients, but not in BE.^1^

Gashi et al from Kosovo conclude that the prevalence of HPI is lower in long segment BE than in short segment BE, but there is no control group.^2^

Irvanloo et al from Iran reported the prevalence of HPI only in BE, but not in controls.^3^

Láng et al from Hungary suggested that HPI is a risk factor for esophageal adenocarcinoma in BO, but it was not supported by detailed data.^4^

Langner et al from Austria and Germany did not report detailed enough data on HPI prevalence in BE or in controls to be eligible for inclusion.^5^

Lee et al from Korea reported 0% prevalence of HPI in BE, but there was no data on HPI prevalence in controls.^6^

O’Connor et al from Ireland reported a HPI prevalence of 62.5% in BE, but there is no control group.^7^

Peitz et al from Germany used the definition of columnar epithelia lined lower oesophagus, which included cases without histological evidence of Barrett’s and H. pylori density was reported instead of prevalence.^8^

Piqué et al from Spain reported data form a large nationwide cross-sectional study, but only 16% of patients had HPI tested.^9^

Rosaida et al from Malaysia reported results from a large cohort of reflux patients, but there was no detailed data on the prevalence of HPI in BE and controls.^10^

Salem et al from the USA did not report any data on BE.^11^

Smith et al from the USA showed that the prevalence of HPI in BE is low, only 1 in 9, but the prevalence of HPI in controls was not reported.^12^

Peng et al in 2010 reported risk of clinically significant endoscopic findings in Barrett’s patients with vs without HPI infection. Raw data on HPI prevalence could not be extracted.

References

1. Agreus L, Talley NJ, Vieth M, et al. Impact of helicobacter pylori on gastroesophageal reflux disease depends on its effects on the gastric corpus mucosa. Gastroenterology. 2015;148:S98.

2. Gashi Z, Ivkovski L, Shabani R, Haziri A, Juniku-Shkololli A. What are Predictive Factors for Developing of Barrett's Esophagus in Patients with Gerd-our Experience. Acta Inform Med. 2011;19:146-148.

3. Irvanloo G, Fallahi B, Ensani F, Azmi M, Morteza A. Endoscopic versus histological diagnosis of Barrett's esophagus: A cross-sectional survey. Polish Journal of Pathology. 2011;62:152-156.

4. Láng J, Bittera B, Varga ZM, et al. Low prevalence of barrett's esophagus and esophageal adenocarcinoma over a 10-year period. United European Gastroenterology Journal. 2013;1:A413-A414.

5. Langner C, Schneider NI, Plieschnegger W, et al. Cardiac mucosa at the gastro-oesophageal junction: indicator of gastro-oesophageal reflux disease? Data from a prospective central European multicentre study on histological and endoscopic diagnosis of oesophagitis (histoGERD trial). Histopathology. 2014;65:81-89.

6. Lee JI, Park H, Jung HY, Rhee PL, Song CW, Choi MG. Prevalence of Barrett's esophagus in an urban Korean population: A multicenter study. J Gastroenterol. 2003;38:23-27.

7. O'Connor HJ, Cunnane K. Helicobacter pylori and gastro-oesophageal reflux disease - A prospective study. Irish Journal of Medical Science. 1994;163:369-373.

8. Peitz U, Hackelsberger A, Günther T, Clara L, Malfertheiner P. The prevalence of Helicobacter pylori infection and the pattern of gastritis in Barrett's esophagus. Digestive Diseases. 2001;19:164-169.

9. Piqué N, Ponce M, Garrigues V, et al. Prevalence of severe esophagitis in Spain. Results of the PRESS study (Prevalence and risk factors for esophagitis in Spain: A cross-sectional study). United European Gastroenterology Journal. 2016;4:229-235.

10. Rosaida MS, Goh KL. Gastro-oesophageal reflux disease, reflux oesophagitis and non-erosive reflux disease in a multiracial Asian population: A prospective, endoscopy based study. European Journal of Gastroenterology and Hepatology. 2004;16:495-501.

11. Salem A, Matthews S, Mori Y, Ibrahim S, Meltzer S, Roland BC. Prevalence and associated risk factors of helicobacter pylori-negative gastritis. American journal of gastroenterology. 2014;109:S40.

12. Smith JG, Li W, Rosson RS. Prevalence, clinical and endoscopic predictors of Helicobacter pylori infection in an urban population. Connecticut Medicine. 2009;73:133-137.

13. Peng S, Xiong LS, Xiao YL, et al. Prompt upper endoscopy is an appropriate initial management in uninvestigated chinese patients with typical reflux symptoms. Am J Gastroenterol. 2010;105:1947-1952.
